# MRI Segmentation and Classification of Human Brain Using Deep Learning for Diagnosis of Alzheimer’s Disease: A Survey

**DOI:** 10.3390/s20113243

**Published:** 2020-06-07

**Authors:** Nagaraj Yamanakkanavar, Jae Young Choi, Bumshik Lee

**Affiliations:** 1 Department of Information and Communications Engineering, Chosun University, Gwangju 61452, Korea; nagarajpy@chosun.ac.kr; 2 Division of Computer & Electronic Systems Engineering, Hankuk University of Foreign Studies, Yongin 17035, Korea; jychoi@hufs.ac.kr

**Keywords:** magnetic resonance imaging, Alzheimer’s disease, deep learning

## Abstract

Many neurological diseases and delineating pathological regions have been analyzed, and the anatomical structure of the brain researched with the aid of magnetic resonance imaging (MRI). It is important to identify patients with Alzheimer’s disease (AD) early so that preventative measures can be taken. A detailed analysis of the tissue structures from segmented MRI leads to a more accurate classification of specific brain disorders. Several segmentation methods to diagnose AD have been proposed with varying complexity. Segmentation of the brain structure and classification of AD using deep learning approaches has gained attention as it can provide effective results over a large set of data. Hence, deep learning methods are now preferred over state-of-the-art machine learning methods. We aim to provide an outline of current deep learning-based segmentation approaches for the quantitative analysis of brain MRI for the diagnosis of AD. Here, we report how convolutional neural network architectures are used to analyze the anatomical brain structure and diagnose AD, discuss how brain MRI segmentation improves AD classification, describe the state-of-the-art approaches, and summarize their results using publicly available datasets. Finally, we provide insight into current issues and discuss possible future research directions in building a computer-aided diagnostic system for AD.

## 1. Introduction

Magnetic resonance imaging (MRI) is used to analyze the anatomical structures of the brain due to its high spatial resolution and ability to contrast soft tissue. It is known that MRI is generally associated with fewer health risks compared to other modalities like computed tomography (CT) and positron emission tomography (PET) [1]. Over the past few decades, tremendous progress has been made in assessing brain injuries and exploring brain anatomy with MRI [2]. Disorders such as Alzheimer’s disease (AD) and multiple sclerosis [3] associated with the brain can be identified using MRI. Tissue atrophy is a popular indicator that is used in diagnosing AD. The segmentation of brain MRI taken at different times is also used to measure structural changes in the brain. Accurate detection and classification of unhealthy tissue and its surrounding healthy structures are also important in the diagnosis of conditions such as AD. A large amount of data is required for more accurate diagnoses. However, it can be challenging for clinicians to analyze large and complex MRI datasets and to extract important information manually. Moreover, due to various inter- or intra-operator variability issues [4], manual analysis of brain MRI is time-consuming and vulnerable to errors. Hence, it is necessary to develop an automated segmentation method to provide accurate results with high confidence. Computerized techniques for MRI segmentation, visualization, and registration have recently been used on large scale datasets to assist clinicians in making qualitative diagnoses.

A number of clinical applications include brain MRI segmentation because it impacts the results of the entire analysis process. A number of classical machine learning-based approaches have been developed for the segmentation of brain tissue types such as gray matter (GM), white matter (WM), and cerebrospinal fluid (CSF). Abnormal tissues of the brain in patients with AD can be segmented in MRI [5]. However, the extraction of the imaging features for such segmentation requires elaborate engineering techniques and specialized expertise. 

The main objective of brain MRI segmentation is to divide the image into well-defined regions, where each region consists of a set of pixels that share the same range of intensities, texture, or neighborhood. Our review discusses the segmentation of these regions from brain images using deep learning techniques. The segmentation of GM, WM, and CSF from brain MRI is challenging due to their tissue intensities, non-uniformity (bias), noise artifacts, and partial volume effect. To overcome these difficulties, several deep learning techniques for brain MRI segmentation have been developed and will be reviewed. We also review various deep learning techniques for the early diagnosis of AD, which is a type of dementia that can cause thinking, memory, and behavioral issues. For the MRI classification of AD, significant patterns from raw data are considered, and these patterns are grouped into different categories based on their characteristics. Significant advances in imaging technology have led to the development of different applications of image segmentation and classification. Brain segmentation plays an important role and can be a building block for AD diagnosis. Semantic segmentation techniques with Freesurfer [6] can predict the volume of the brain from MRI scans of patients with AD [7]. Unsupervised hierarchical segmentation methods for AD diagnosis can detect homogeneous regions and separate them from coarse to finer levels, providing more flexibility for multi-level analysis than single-level semantic segmentation [8,9]. Furthermore, precuneus atrophy and the hippocampus are the most sensitive biological indicators of AD, particularly at an early stage [10]. The precuneus, an area of the posteromedial cortex, has recently received significant attention in functional neuroimaging studies. Precuneus atrophy observed in the AD group suggests that MRI volumetric assessment of precuneus volume, in addition to the hippocampal volume, might be a useful radiological index for the diagnosis of AD.

**Figure 1:**
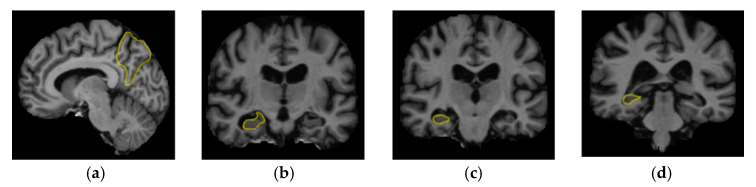
The regions of interest (outlined using yellow color) illustrating the boundaries of the left precuneus (**a**) in the sagittal plane and right hippocampus (**b**) head (**c**) body and (**d**) tail.

Figure 1 shows the boundaries of the left precuneus in the sagittal plane, and the entire anterior-posterior extent of the hippocampus (including its head, body, and tail) outlined on coronal MR images. These anatomical boundaries are used for volumetric measurements in the precuneus and hippocampus.

Traditional machine learning approaches have relatively lower performance with larger amounts of input data. It can be challenging to detect brain abnormalities correctly and to find a solution for the automatic segmentation of brain structures. Such challenges mainly arise from the changes in settings for the acquisition of MRI scans, fluctuations in the appearance of pathology, normal anatomical variations in brain morphology, and imperfections in image acquisition. The limitations of traditional machine learning methods can be overcome by deep learning-based approaches. Moreover, deep learning can also be used to perform quantitative analysis of brain MRI through the self-learning of features, by which new features can be recognized. Deep learning has been acquiring substantial attention in various medical image analyses [11], such as computer-aided diagnosis of breast lesions [12], pulmonary nodules [13], and histopathological diagnosis [14].

This paper aims to provide an outline of progressive deep learning methods in the area of MRI segmentation of normal and abnormal (AD) brain tissue. Furthermore, we analyze existing problems in the segmentation of brain MRI that can be overcome with deep learning. In summary, the main objectives of this review are to:

- Provide an overview of the current deep learning approaches for brain MRI segmentation and classification of AD.
- Identify the application challenges in the segmentation of brain structure MRI and classification of AD.
- Show that MRI segmentation of the brain structure can improve the accuracy of diagnosing AD.

The rest of the paper is organized as follows. A brief overview of publicly available brain MRI datasets, followed by a brain MRI analysis, is presented in Section 2. An overview of convolutional neural networks (CNN) architecture, segmentation of brain structure MRI using deep learning, and how segmentation improves the classification of AD are described in Section 3. The evaluation measures for brain MRI segmentation is presented in Section 4. Finally, we conclude with a general discussion and explore future directions in the field of brain MRI segmentation.

## 2. MRI Dataset for Brain Analysis

The data evaluation framework of three-dimension (3D) cross-sectional brain MRI is used to classify patients with AD and to segment brain tissue types (CSF, GM, and WM). Publicly available datasets such as open access series of imaging studies (OASIS) [15], Alzheimer’s disease neuroimaging initiative (ADNI) [16], medical image computing and computer-assisted intervention (MICCAI) [17], and internet brain segmentation repository (IBSR) [18] are popularly used for segmentation of brain MRI and AD diagnosis. Table 1 shows the details of the OASIS, ADNI, IBSR, and MICCAI datasets. The details of these datasets are described below and then followed by an analysis of brain MRI at various stages.

### 2.1. Public Dataset for Brain MRI

#### 2.1.1. OASIS

The OASIS dataset [15] was created by Washington University, where the Alzheimer’s Disease Research Centre manages a large amount of longitudinal and cross-sectional brain MRI data from non-demented and demented subjects. The longitudinal dataset contains multiple scans of each subject over a period of time, and the cross-sectional category includes the details of 416 subjects aged between 18 and 96 years. The risk factor of AD can be measured with the clinical dementia rating (CDR) [19] and mini-mental state examination (MMSE) [20]. The subjects are examined with risk factors as non-demented for CDR 0, very mild dementia for CDR 0.5, mild dementia for CDR 1, and moderate dementia for CDT 2, as indicated in [15]. 

#### 2.1.2. ADNI

AD usually observed in elderly individuals can be diagnosed using the ADNI dataset [16], which contains details of MRI scans for 843 subjects with scanner intensity fields ranging from 1.5 T to 3 T. It is observed that patients with lower cognitive abilities including thinking and memory loss are associated with mild cognitive impairment (MCI). They also have a high risk of transforming to AD or any other types of dementia and are grouped separately from AD. 

#### 2.1.3. IBSR

The IBSR dataset [18] is used to evaluate and develop segmentation techniques for brain images. This dataset provides manually guided expert segmentation results along with the MRI data. It consists of 20 real T1-Weighted (T1-W) MRI with manually guided expert segmentation results, referred to as the ground truth. In addition, each MRI volume contains around 60 coronal T1-W slices with a 3.1 mm resolution (slice gap between successive slices) and 18 cortical T1-W slices with a resolution of 1.5 mm. The subject volumes of this dataset have dimensions of 256 × 256 × 128 pixels and different voxel spacings: 0.84 × 0.84 × 1.5 mm^3^ 0.94 × 0.94 × 1.5 mm^3^, and 1.0 × 1.0 × 1.5 mm^3^. In addition, Massachusetts General Hospital has provided manual segmentation of 32 noncortical structures.

#### 2.1.4. MICCAI

The MICCAI-2012 dataset [17] consists of 35 T1-w MRI volumes and manual segmentation of 134 structures obtained from Neuromorphometrics, Inc., Scotts Valley, CA, USA. It is mainly used for the segmentation of tissue, tumor, and structure. This dataset started with 80 real and synthetic cases in 2012. The size of the training and testing data has increased over the years. The MICCAI 2012 challenge in multi-atlas labeling is used for sub-cortical structure segmentation.

### 2.2. Pre-Processing for Brain MRI Analysis

**Figure 2:**
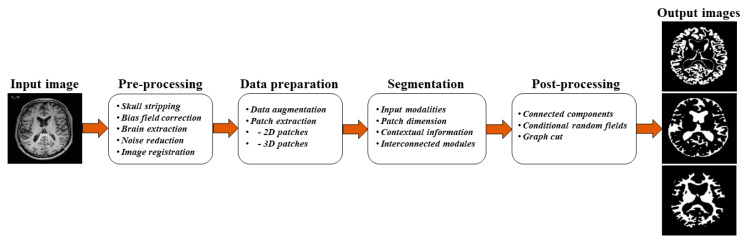
General pipeline for brain MRI analysis.

**Figure 3:**
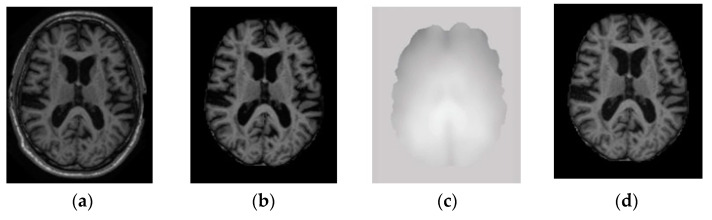
Pre-processing of MRI. (**a**) T1-W original MRI, (**b**) Brain tissue image after removal of the nonbrain structure, (**c**) The bias field, (**d**) Brain tissue image after bias field correction.

Figure 2 illustrates a typical pipeline for the segmentation stages of brain MRI analysis, conventionally proposed in the literature from a top-level perspective. The overall pipeline consists of four main stages, including pre-processing, data-preparation, segmentation, and post-processing. Different pre-processing tasks are required after acquiring MRI so that the images can be used for the segmentation of various tissue types of the brain. Figure 3 shows examples of brain MRI in pre-processing. The pre-processing for MRI includes brain extraction, bias field correction, and image registration.

Brain extraction: Brain MRI shows tissue as well as parts of the head, eye, fat, spinal cord, and skull [21]. Skull stripping to extract brain tissue from the non-brain tissue needs to be performed to identify the voxels as brain or non-brain. The output of skull stripping can be a new image only with brain voxels or a binary value assigning value 1 for brain voxels and value 0 for the rest of the tissue. In general, brain voxels include the brain stem, CSF, GM, and WM of the cerebral cortex, subcortical structures, and cerebellum, whereas non-brain voxels include the scalp, matter, eyes, bones, dura, skin, muscles, and fat [22]. An example of an original T1-W brain MRI and corresponding skull stripped output is shown in Figure 3a,b, respectively. 

Bias field correction: Image contrast due to magnetic field inhomogeneity [23] can be fine-tuned with the help of bias field correction. The bias field depends on the strength of the magnetic field and is almost negligible when the MRI is performed at 0.5 T. However, when the magnetic field strength is 1.5 T, 3 T, or larger, it is considered as strong and can impact the analysis of the MRI. Figure 3c shows the bias field, and Figure 3b,d shows the MRI before and after bias field correction, respectively. 

Noise reduction: Noise reduction is the process of lowering locally variant Rician noise noticed in MRI [23]. This is considered to be less critical for classification applications using deep learning [24].

Image registration: Image registration is mainly used to convert the alignment of the images from spatial to common anatomical spaces [25] and has two types, inter- and intra-patient image registration. The inter-patient image registration is used to standardize MRI onto standard stereotaxic spaces, whereas intra-patient registration aids in aligning MRI of different sequences (T1- and T2-W images) to obtain multi-channel characterization for each position within the brain. After pre-processing, data preparation is performed with data augmentation or patch-based strategies from the input volumes. Then, segmentation or classification according to the objective of the analysis is performed based on input modalities and patch dimensions. Finally, the results obtained could be refined by choosing the largest groups only or smoothing regions.

## 3. Review of Brain MRI Segmentation and Diagnosis

In this section, we provide a comprehensive literature review on the segmentation of the brain structure and classification of brain MRI for diagnosing AD. Moreover, we briefly discuss CNN architecture, followed by the segmentation of brain structures using deep learning techniques. Then, the classification of AD using deep learning is presented. Finally, we discuss how the segmentation of brain MRI improves the classification accuracy of MRI for AD.

### 3.1. Overview of CNN Architecture

Deep learning refers to neural networks with a deep number of layers (usually more than five) that extract a hierarchy of features from raw input images. Traditional machine learning algorithms [26,27,28,29,30] extract features manually, whereas deep learning extracts complex, high-level features from the images and trains a large amount of data, thus resulting in greater accuracy. Owing to significantly increased GPU processing power, deep learning methods allow us to train a vast amount of imaging data and increase accuracy despite variations in images.

Various applications such as image segmentation, genotype/phenotype detection, classification of diseases, object detection, and speech recognition utilize different deep learning approaches. Some of the popular deep learning algorithms include deep Boltzmann machines, CNNs, stacked auto-encoders, and deep neural networks. CNNs are prominently used for image segmentation and classification. Even though CNNs were first introduced in 1989 [31], they received more attention after observing their excellent performance in the ImageNet [32] Competition in 2012 [33]. It is reported that, by applying CNN on a dataset with millions of images with 1000 various classes, the error rate can be reduced to half, compared to earlier best computing approaches [34]. CNN architecture is increasingly complex, with a large number of layers, including neurons with millions of weights and a large number of connections between different neurons, indicating that the computational complexity of CNN architecture is high.

**Figure 4:**
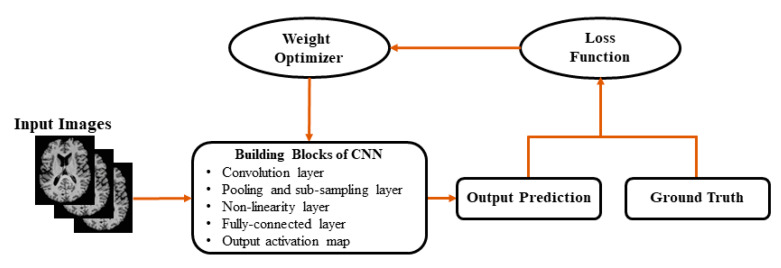
Generally used architecture of convolutional neural networks (CNNs).

Figure 4 shows the basic block diagram of CNN, which consists of layers of convolution, pooling, activation function, and fully connected layers with each layer performing specific functions. Input images are convolved across the kernel by the convolutional layer to produce feature maps. In the pooling layer, as the value transferred to the successive layer, the results obtained from preceding convolutional layers are downsampled using the maximum or average of the specified neighborhood. The most popular activation functions are the rectified linear unit (ReLU) [14] and the leaky ReLU [14], which is a modification of ReLU. The ReLU transforms data nonlinearly by clipping off negative input values to zero and passing positive input values as output. The results of the last CNN layer are coupled to loss function (e.g., scores are normalized into a multinomial distribution over labels by cross-entropy loss) to provide a forecast of the input data. Finally, network parameters are obtained by decreasing the loss function between prediction and ground truth labels along with regularization constraints. In addition, weights of the network are updated at each iteration (e.g., using stochastic gradient descent) using backpropagation until the convergence.

Table 2 shows various segmentation strategies using single-modality, multi-modality, semantic-wise, patch-wise and cascaded using the CNN architecture. Single-modality refers to a single source of information and is adaptable to different scenarios. In contrast, multi-modality utilizes multi-source information and provides exact localization, highlighting any pathognomonic changes and metabolic activity of the target tissue in the case of positron emission tomography (PET). Semantic-wise approaches link each pixel of an image with its class label. Segmentation labels are mapped with the input image so that it minimizes loss function. This allows segmentation maps to be generated for any image size. The computational complexity of this method is much lower than other approaches [34]. This principle in segmentation is used for most of the state-of-the-art approaches [35,36,37], as will be described in Section 3.2. The patch-wise approach takes small patches from high-resolution images. That is, the input images are split as a number of local patches and trained. This can result in better local information by predicting the information for an individual patch. Moreover, the model can be trained with local details in patch-wise approaches but does require higher computational complexity. Cascaded CNN is characterized by the first network, which provides the first classification, and the second network receiving the outputs of the first network (as input) to refine the classification. Cascaded CNN achieves competitive results in comparison with other CNN approaches. Despite the success in segmentation performance using deep learning, there are also several problems and limitations.

Challenges in brain MRI analysis for segmentation and classification using deep learning:

- Deep learning used in big data analytics: The major challenge lies in the difficulty of obtaining a large enough dataset to train and improve the accuracy of the model properly. Deep learning faces difficulties in dealing with the volume (high-dimensional decision space, and a large number of objectives), variety (modeling using heterogeneous data and knowledge transfer between problems), variability (robustness over time and online knowledge acquisition) and veracity (noisy fitness evaluations and surrogate-assisted optimization) of big data [50]. To overcome this problem, the author in [51] suggested various optimization techniques such as global optimization, which reuse the knowledge extracted from the vast amount of high dimensional, heterogeneous, and noisy data. On the other hand, complex optimization techniques provide efficient solutions by formulating new insights and methodologies for optimization problems that take advantage of using deep learning approaches when dealing with big data problems. The traditional machine learning approaches show better performance with less input data. As the amount of data increases beyond a certain critical point, the performance of traditional machine learning approaches becomes steady, whereas deep learning approaches tend to increase [51]. Deep learning architectures, such as deep neural networks, deep belief networks, and recurrent neural networks, have been applied to research fields including medical image analysis, bioinformatics, and computer vision, where they often produce impressive results, that are comparable to and superior to human experts in some cases.
- Scalability of deep learning approaches: The scalability of deep learning needs to consider not only accuracy but several other measures regarding computational resources. Scalability plays a vital role in deep learning. As data expands in terms of variability, variety, veracity, and volume, it becomes increasingly difficult to scale computing performance using enterprise-class servers and storage in line with the increase. Scalability can be achieved by implementing deep learning techniques on a high-performance computing (HPC) system (super-computing, cluster, sometimes considered cloud computing), which offers immense potential for data-intensive business computing [50]. The ability to generate data, which is important where data is not available for learning the system (especially for computer vision tasks, such as inverse graphics).
- Multi-task, transfer learning, or multi-module learning: Learning simultaneously from several domains or with various models is one of the significant challenges in deep learning. Currently, one of the most significant limitations to transfer learning is the problem of negative transfer. Transfer learning only works if the initial and target problems are similar enough for the first round of training to be relevant. If the first round of training is too different, the model may perform worse than if it had never been trained at all. There are no clear standards on what types of training are sufficiently related, or how this should be measured.

### 3.2. Segmentation of Brain MRI Using Deep Learning

To perform a quantitative analysis of the brain tissue, and large-scale study of intracranial volume, accurate automated segmentation of brain structures such as GM, WM, and CSF in MRI is crucial. The traditional approaches used for the segmentation of brain tissues include the Atlas-based approach and pattern recognition approach: Atlas-based approaches [52,53,54,55] match intensity information between an atlas and target images. Atlas-based and registration are among the methods which are widely used for human brain segmentation [56,57,58] but do not provide robust results for small and highly variable structures like the hippocampus, due to limitations in registration and variability in reliable ground truth data. In pattern recognition approaches [37,59,60], brain tissues are classified based on the set of local intensity features. Recently, hippocampal atrophy has been proposed as a biomarker of AD [61,62]. The hippocampus is a part of the brain’s limbic system surrounded by different kinds of tissue. A number of studies have shown that a lower hippocampal volume is observed in patients with AD [63,64]. Hence, MRI segmentation of the hippocampus could have practical value in clinical applications [65]. However, segmentation for the hippocampus in MRI is challenging due to its small size, partial volume effects, anatomical variability, low contrast, low signal-to-noise ratio, indistinct boundary, and proximity to the Amygdaloid body. Furthermore, manual segmentation requires time-consuming expert analysis. A recent study shows that segmenting the hippocampus, thresholding or region growing using conventional methods do not achieve acceptable results [66]. Wang et al. [67] proposed a region growing algorithm based on seed, which is simple and effective, yet fails to obtain promising results because of the unclear edges of the hippocampus [68]. 

Table 3 shows the list of studies on the segmentation of brain tissues based on CNN and also the type of public dataset used in the analysis. In addition, the summary of segmentation strategies with their image dimension followed by classifiers used in the CNN architecture are described. Furthermore, an overview of existing methods based on deep learning for the segmentation of brain tissues and anatomical segmentation (e.g., the hippocampus) is summarized in Table 4.

Challenges in brain MRI segmentation:

- Large variations in brain anatomical structures due to phenotypes, age, gender, and disease. It is difficult to apply one specific segmentation method to all phenotypic categories for reliable performance [77].
- It is challenging to process cytoarchitectural variations, such as gyral folds, sulci depths, thin tissue structures, and smooth boundaries between different tissues. This can result in confusing categorical labeling for distinct tissue classes. This is difficult even for human experts.
- The low contrast of anatomical structure in T1, T2, and FLAIR modalities results in low segmentation performances.
- Manual segmentation for brain MRI is laborious, subjective, and time-consuming, and requires sophisticated knowledge of brain anatomy. Thus, it is difficult to obtain enough amount of ground truth data for building a segmentation model.
- The noisy background in the ordinary image for segmentation is challenging because it is hard to assign an accurate label to each pixel/voxel with learned features.
- The segmentation of the hippocampus, which is one of the most important biomarkers for AD, is difficult due to its small size and volume [65], as well as its anatomical variability, partial volume effects, low contrast, low signal-to-noise ratio, indistinct boundary and proximity to the Amygdaloid body.

### 3.3. Brain MRI Classification of AD Diagnosis Using Deep Learning

The segmentation of brain MRI is carried out to eliminate unnecessary details and to locate relevant objects from the processed images. The detailed analysis of the tissue structures from the segmented MRI leads to a more precise classification of specific brain disorders such as AD. AD is more common in elderly individuals, and it is considered to be a common form of dementia. Patients with AD suffer from the degradation of cognitive abilities over time. In advanced cases, patients struggle with activities of daily life, ultimately resulting in an inability to self-care. In this disease, nerve cells and tissues of the human brain are affected. Initially, the frontal lobe of the cerebral cortex, which helps in planning, thinking, and remembering, and the hippocampus, which plays a crucial role in the development of new memories, can be affected. Although vulnerability to AD increases in those over the age of 65 years, AD is not solely associated with old age [78]. A recent study [79] estimates that more than 90 million people will have AD by 2050. Despite considerable research to discover treatments for AD and halt or delay its progression, so far, there have not been promising results [80].

Table 5 summarizes the classification methods for the diagnosis of AD using CNN architectures on the public datasets (OASIS and ADNI). Furthermore, considerable research efforts have been made for the classification of AD. The applications and key features of the methods are described in Table 6. 

Challenges in the diagnosis of AD:

- The automatic classification of AD is quite challenging due to the low contrast of the anatomical structure in MRI. The presence of noisy or outlier pixels in MRI scans due to various scanning conditions may also result in a reduction of the classification accuracy.
- The major challenge in AD is that it is difficult to make long-term tracking and investigation of the patient’s pathology. Thus, it is not easy to track the transition of AD status. In the ADNI dataset [16], there are only 152 transitions in total out of the entire dataset of 2731 MRIs. Due to the lack of the MRIs in terms of tracking the transition of AD status, it is likely for the model to overfit without generalizing distinctions between different stages of AD.
- It is well known that AD is not only diagnosed from clinical stages of brain MRI, but also occurs through abnormal amyloid β peptide (Aβ) and tau (*τ*) protein activity around neurons and their temporal relationship with the different phases of AD in different stages. The factors mentioned above should be considered as multi-modal biomarkers as well as brain MRI. Thus, complexity during the process of treating AD is due to diverse factors regulating its pathology.
- Data multimodality in the diagnosis of AD
  ✓ Since neuroimaging data (i.e., MRI or PET) and genetic data (single nucleotide polymorphism (SNP)) have different data distributions, different numbers of features and different levels of discriminative ability to AD diagnosis (e.g., SNP data in their raw form are less effective in AD diagnosis), simple concatenation of the features from multimodality data will result in an inaccurate prediction model [105,106] due to heterogeneity.
  ✓ High dimensionality issue: One neuroimage scan normally contains millions of voxels, while the genetic data of a subject has thousands of AD-related SNPs.

### 3.4. The Segmentation of Brain MRI Improves the Classification of AD

It is known that AD is the major cause of dementia for most Caucasians [107]. An important pathological characteristic of AD is diffuse brain atrophy, which includes atrophy of the cerebral cortex, enlargement of the ventricles, and atrophy of the medial temporal lobe (MTL) structures such as the hippocampus [108,109]. It is reported that AD pathology is associated with GM and WM tissues, and it was discovered that abnormalities of these tissues are highly correlated with cognitive decline [110,111,112]. To more clearly capture how AD evolves, neuropsychological and anatomical information from the patient needs to be examined at different transitional phases of the disease. From these aspects, the populations suffering from MCI have a high chance of converting disease status from MCI to AD. The results in [113,114] show that the risk among individuals converting from MCI to AD is significantly increased as compared to normal individuals. Among various types of MCIs, the amnesic form (aMCI) is commonly found in most individuals. As the highest annual incidence of conversion from aMCI to AD is observed [113,114], aMCI is considered to be the prodromal phase of AD. In previous studies [108,115,116,117], the atrophy of MTL structures, such as the hippocampus, amygdala, and entorhinal and parahippocampal cortices, increases with progression of the disease. Considerable research effort has focused on the segmentation of the hippocampus to evaluate the volume or shape of the brain [109,118,119,120,121,122,123,124,125,126,127,128,129,130]. Cortical thickness and GM tissue maps [131] have also been shown to have high predictive value in the diagnosis of AD [131]. In addition, studies on group differences based on voxel-based morphometry (VBM) [132], deformation-based morphometry (DBM) [133], or tensor-based morphometry (TBM) [134] were investigated. The VBM is a spatially specific and unbiased method of analysis of MRI, showing the regional GM and WM volume at a voxel scale [135]. This method has been applied to AD and MCI, reflecting patterns of GM irregularities that are suitable for the clinical phase of the disease, and also predict the risk of conversion from MCI to AD [136,137,138]. The presence and extent of WM atrophy are more problematic, and often other MRI methods are used to analyze the presence of microscopic tissue impair in AD [139]. Furthermore, the CSF and structural imaging markers are considered to be primary indicators amended to the current diagnostic criteria of MCI and AD [116]. The CSF markers (Ab42, t-tau, and p-tau) with the volumes of the hippocampus and lateral ventricles, can be combined for distinguishing between HC and MCI, while the CSF Ab42 marker with the shape of the hippocampus and lateral ventricles is a good combination for identifying MCI and AD [140]. Recently, deep learning approaches showed better performance for the automatic segmentation of the hippocampus and classification of AD. The deep CNN model is constructed to learn features of the 3D patches extracted based on the hippocampal segmentation results for the classification task [141]. A multi-task deep learning (MDL) method was proposed for joint hippocampal segmentation and clinical score regression using MRI scans [142]. Choi et al. [143] shown that deep CNN based biomarkers are strongly correlated with future cognitive decline using PET.

**Figure 5:**
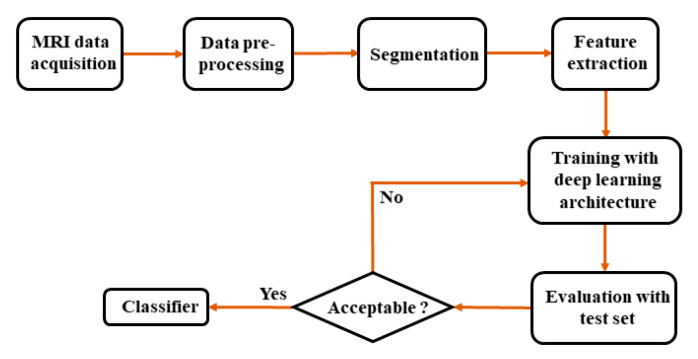
The overall block diagram of AD diagnosis.

The overall block diagram of the diagnosis of AD usually adopted in conventional methods is depicted in Figure 5. The first stage is to acquire the slices from the MRI. This is followed by data preparation with pre-processing, where non-relevant information is removed, and the data are reoriented for more straightforward interpretation. Segmentation using deep learning is performed on the pre-processed data to extract the attributes from brain MRI. Based on attributes such as surface area, the center of gravity, average intensity, and standard deviation, the classifier predicts the output based on prior knowledge using deep learning architecture and decides whether the patient is developing AD or not.

## 4. Evaluation Metrics for Brain MRI Segmentation

In medical image analysis, validation and quantitative comparison are common problems of different segmentation methods. For validation, ground truth data is required for the comparison of segmented output. In a real scenario, there is not enough amount of ground truth data available for the assessment of acquired data in humans. Thus, the ground truth data of patients are manually generated by experts after the MRI acquisition. Although this is the only way to validate the real patient’s MRI data, this validation must be carefully considered because manual segmentation is prone to errors, highly subjective, and difficult to reproduce (even by the same expert) [144]. To overcome these limitations, several alternate validation methods with software simulation and phantoms have been proposed. Software simulation generates artificial images that simulate the process of real acquisition. Likewise, ground truth is known, and the various acquisition parameters, imaging artifacts can be managed and analyzed independently. This type of validation requires less effort and is more flexible to implement. However, the software simulation method does not consider all factors which might impact real image acquisition, and the images obtained for software simulation are only an approximate estimation of the real images. MRI scanners are used to produce phantom images that appear to be more realistic compared to the ones obtained from software simulations. However, phantom images are not flexible. Additionally, software simulations and phantom imaging results are expensive and time-consuming. To evaluate the overlap between the predicted brain MRI and the given ground truth image, various similarity measures were used [145]. 

The most well-known evaluation measure is the Dice coefficient [146]. The quality of two binary label masks can be compared with this volume. Let us define that $D_{P}$ is the mask by the human evaluator and $D_{Q}$ is the mask generated by a segmented algorithm, the Dice overlap is then evaluated as Equation (1).

(1) $$Dice\;(D_{P},D_{Q})=\frac{2\left|D_{P}\cap D_{Q}\right|}{\left|D_{P}\right|+\left|D_{Q}\right|}$$

where |.| represents the number of voxels. The overlap measure has values in the range of [0, 1], with 0 indicating no match between the two masks and 1 indicating that the two masks are identical. The Jaccard index [147] is also used as a similarity measure for the comparison of two binary label masks, and it is expressed as Equation (2).

(2) $$Jaccard\;Index\;(D_{P},D_{Q})=\frac{\left|D_{P}\cap D_{Q}\right|}{\left|D_{P}\right|+\left|D_{Q}\right|-\left|D_{P}\cap D_{Q}\right|}$$

The ratio of true positives to the sum of true and false positives is known as the positive predicted value (PPV). It is also called as precision and expressed as Equation (3).

(3) $$PPV(D_{P},D_{Q})=\frac{\left|D_{P}\cap D_{Q}\right|}{\left|D_{P}\cap D_{Q}\right|+\left|D_{P}^{c}\cap D_{Q}\right|}$$

The ratio of true positives to the sum of true and false positives is known as the positive predicted value (PPV). It is also called as precision and expressed as Equation (3). The ratio of true positives to the sum of true positives and false negatives is known as the true positives rate and is calculated as Equation (4).

(4) $$TPR(D_{P},D_{Q})=\frac{\left|D_{P}\cap D_{Q}\right|}{\left|D_{P}\cap D_{Q}\right|+\left|D_{P}\cap D_{Q}^{c}\right|}$$

The ratio of true positives to the sum of true positives and false negatives is known as the lesion true positive rate (LTPR). Considering the list of lesions,$\;L_{P}$, as the 18-connected components of $D_{P}$ and similarly,$\;L_{Q}$, as the 18-connected components of $D_{Q}$. It is expressed as Equation (5).

(5) $$LTPR(D_{P},D_{Q})=\frac{\left|\;L_{P}\cap L_{Q}\right|}{\left|\;L_{P}\cap L_{Q}\right|+\left|\;L_{P}\cap L_{Q}^{c}\right|}$$

where $\left|\;L_{P}\cap L_{Q}\right|$ refers to the overlap between a connected component of $D_{P}$ and $D_{Q}$. It is shown that the human evaluator and segmentation algorithm have identical lesions. Furthermore, the lesion wise ratio of false positives to the sum of false positives and true negatives is known as the lesion false positive rate (LFPR), which can be expressed as Equation (6).

(6) $$LFTR(D_{P},D_{Q})=\frac{\left|L_{P}^{c}\cap L_{Q}\right|}{\left|L_{P}^{c}\cap L_{Q}\right|+\left|L_{P}^{c}\cap L_{Q}^{c}\right|}$$

where $L_{R}^{c}$ is the 18-connected components of $D_{P}^{c}.$ The absolute difference in volume divided by the true volume is called the absolute volume difference (AVD) and is expressed as Equation (7).

(7) $$AVD(D_{P},D_{Q})=\frac{\mathrm{Max}\;\left(\left|D_{P}\right|,\left|D_{Q}\right|\right)-\mathrm{Min}\;\left(\left|D_{P}\right|,\left|D_{Q}\right|\right)}{\left|D_{P}\right|}$$

The average symmetric surface distance (ASSD) is the average of the distance (in millimeters) from the lesions in $D_{P}$ to the nearest lesion identified in $D_{Q}$ plus the distance from the lesions in $D_{P}$ to the nearest lesion identified in $D_{Q}$. It is described as Equation (8).

(8) $$ASSD\;(D_{P},D_{Q})=\frac{\sum_{p\in \;L_{P}}d\left(r,\;L_{Q}\right)+\sum_{q\in \;L_{Q}}d\left(r,\;L_{P}\right)}{2}$$

where $d\left(r,\;L_{Q}\right)$ is the distance from the lesion r in $\;L_{P}$ to the nearest lesion in $L_{Q}$. The value of 0 refers to $D_{P}$ and $D_{Q}$ being identical. The detail about similarity measures for validation and evaluation in medical image analysis is found in [132]. Table 7 shows the summary of the validation measures of brain segmentation and their mathematical formulation concerning the number of true positives (TP), false positives (FP), and false-negative (FN) at voxel and lesion levels (TPL, FPL, and FNL).

## 5. Discussion and Future Directions 

Two-dimensional (2D) networks are used in most of the works, which are summarized in Table 3. Table 8 displays information about the various architectures in relation to their performance. The best generalization can be achieved with the help of optimized layers of architecture and choosing the better hyper-parameters with advanced training procedures.

Table 8 shows the summary of the results in the reviewed paper using deep learning approaches for brain structure segmentation results reported in the recent investigations in DSC and JI values as evaluation metrics. Per the results in Table 8, Wachinger et al. [71] show better results in terms of DSC compared to the other methods using the MICCAI dataset because the author introduced explicit within-brain location information through Cartesian and spectral coordinates to help the classifier to discriminate one class from another. Furthermore, Dolz et al. [70] implemented a 3D fully convolution network architecture which was used to segment sub-cortical structures in the brain MRI and the result outperforms other methods in terms of DSC in IBSR datasets. However, it is noted that these results are not able to be accurately compared because these studies used different datasets and experimental conditions. 

It is necessary to have a universal architecture to provide a good representation of the underlying input image without suffering from significant over-fitting to enable accurate comparison. In previous studies on AD-related brain disease diagnosis, MRI-based computer-aided diagnosis methods [148] usually contain three fundamental components, i.e., pre-determination of ROIs, extraction of imaging features, and construction of classification models. Depending on the scales of the pre-defined ROIs in MRI for subsequent feature extraction and classifier construction, these methods can be further divided into three categories, i.e., voxel-level, region-level, and patch-level morphological pattern analysis methods. Specifically, voxel-based methods [149,150] attempt to identify voxel-wise disease-associated microstructures for AD classification. In contrast, region-based methods [87,142] extract quantitative features from pre-segmented brain regions to construct classifiers for identifying patients from HCs. To capture brain changes in local regions for the early diagnosis of AD, patch-based methods [128,129] adopt an intermediate scale (between the voxel-level and region-level) of feature representations for MRI to construct classifiers. Table 9 shows the summary of the various state-of-the-art methods for the classification of AD vs. HC and pMCI (progressive MCI) vs. sMCI (stable MCI) on MRI datasets. In contrast to the studies that only used MRI datasets of ADNI-1, Lian et al. [95] evaluated the method on larger cohorts composed of 1,457 subjects from both ADNI-1 and ADNI-2. The authors used a more challenging evaluation protocol (i.e., independent training and testing sets) and obtained competitive classification performance, especially for pMCI vs. sMCI conversion prediction. The results in [85] outperform other methods in AD vs. HC classification because transfer learning can be used to enhance generality of the features capturing the AD biomarkers with a three stacked 3D convolutional autoencoder network, which is pre-trained on the ADNI datasets. The extracted features are used as AD biomarkers in lower layers of a 3D CNN network. Similar performances were observed in [109], where authors propose a combined use of AlexNet and the data permutation scheme, and it outperforms other methods in AD classification owing to spatial correlation features generated by using localized convolution kernels in CNN and more informative slice selection that trains AlexNet more effectively.

Evaluating the model on different datasets is one of the suggested practice methods. Some works [37,43,151] have validated the models on three or more different public datasets and reported the results. These kinds of practices can be used to make the model robust and can also be applied to a dataset that consists of data from different imaging modalities and MRI scanners [152]. It is exceedingly challenging to train deep CNNs with low-resolution MRI, and the need for a short prediction time makes it impossible to train deep CNNs [115]. Therefore, training such networks can be performed by designing a method with faster convolution. The computation speed of CNN was improved by using FFT algorithms and fast matrix multiplication methods [153,154], yet the training algorithms of deep CNNs can be improved using variants of SGD [155] and their parallelized implementations. It is expected to improve the performance of deep CNNs with the high optimization method using emerging new algorithms with less or no hyper-parameters, which constitute one of the major bottlenecks for most users. 

One of the main contributors to the steep rise of deep learning has been the widespread availability of GPU. GPU has parallel computing engines that perform with execution threads larger than central processing units (CPUs). It is known that deep learning on GPUs is typically 10–30 times faster than on CPUs [156]. Another driving force behind the popularity of deep learning methods is the wide availability of open-source software packages. Deep learning libraries such as Caffe [157], PyTorch [158], Tensorflow [159], and Theano [160] are most often used. Summaries of the hardware and software requirements for brain MRI segmentation and classification of AD using deep learning approaches are described in Table 10.

According to recent research, deep learning is promising for the analysis of brain MRI and can overcome the issues associated with the earlier state-of-the-art machine learning algorithms. Brain MRI analysis using computer-aided techniques has been challenging because of its complex structure, irregular appearance, imperfect image acquisition, non-standardized MR scales, imaging protocol variations, and presence of pathology. Hence, more generic methods using deep learning are preferable to manage these vulnerabilities.

Even though deep learning techniques in brain MRI analysis have great potential, there are still some limitations. It does not display competitive results on relatively small datasets, whereas it outperforms other methods on large datasets like ImageNet [161]. Several studies have shown that most of the methods consistently achieve better results when the size of the training datasets is increased [162,163]. There has been an increasing demand for large-scale datasets so that deep learning techniques can be used more efficaciously. It is challenging to acquire a large amount of brain MRI data due to legal and privacy issues. Therefore, it is necessary to develop a deep learning method using many different brain MRI datasets. One solution is to augment the data from the existing dataset. For this, random transformations such as translation, flipping, deformation, and rotation called data augmentation can be applied to the original data to increase the size of the dataset and are applied in deep learning methods. Many studies have shown that data augmentation leads to benefits that introduce random variations to the original data and reduce overfitting [33,150]. Furthermore, supervised learning techniques construct predictive models by learning from a large number of training examples, where each training example has a label indicating its ground-truth output. Although current techniques have achieved great success, it is difficult to get strong supervision information like fully ground truth labels due to the high cost of data labeling in many applications. In circumstances in which there are difficulties in obtaining large brain MRI datasets with ground truth annotations, deep learning techniques should work with weak supervision, which can be trained on small datasets. Deep weakly-supervised learning models can be a solution to identify diseases in brain MRI without the need for a large number of ground truth annotations. These models allow us to simultaneously classify brain MRI, yielding pixel-wise localization scores, thereby identifying the corresponding regions of interests (ROIs). Transfer learning could be used to share well-performing deep learning models, which are trained on normal and pathological brain MRI data, among the brain imaging research community. The generalization ability of these models could then be improved across datasets with less effort required.

## 6. Conclusions

In medical imaging, the advancement of computational intelligence, deep learning, and computer-aided detection has attracted much attention in brain segmentation and AD diagnosis. Although deep learning methods have a high impact on the quantitative analysis of brain MRI, there is still difficulty in finding a robust, generic method. Pre-processing initialization and post-processing can affect the performance of deep learning techniques. Here, we reviewed the-state-of-the-art studies of brain structure and classification of brain MRI for the diagnosis of AD. Moreover, we discussed how brain structure segmentation improves the classification performance of AD. The segmentation for brain MRI helps to facilitate the interpretation and classification of AD. Brain MRI segmentation can be challenging work due to the images having a noisy background, partial volume effect, and low contrast. Furthermore, the automatic classification of AD is quite challenging due to the low contrast of the anatomical structure in MRI. To overcome these difficulties, various methods for segmentation have been proposed, with varying complexity. These methods have resulted in more accurate results in the past few decades. The segmentation of the brain structure and classification of AD using deep learning approaches has gained attention due to the ability to provide efficacious results over a large-scale data set as well as to learn and make decisions on its own.

## Figures and Tables

**Table 1:** Details of the OASIS, ADNI, IBSR, and MICCAI datasets.

Dataset	Class	# of Subjects	Sex		Age		MMSE		# of MRI Scans
			M	F	Mean	Std	Mean	Std	
OASIS	AD	100	41	59	76.76	7.11	24.32	4.16	100
	HC	316	119	197	45.09	23.11	29.63	0.83	316
ADNI	AD	192	101	91	75.3	7.5	23.3	2.1	530
	MCI	398	257	141	74.7	7.4	27.0	1.8	1126
	HC	229	120	109	75.8	5.0	29.1	1.0	877
IBSR	HC	18	14	4	71	-	-	-	18
MICCAI	HC	35	-	-	-	-	-	-	35

**Table 2:** Categorization of segmentation methods using CNN architecture on brain MRI.

Strategies	Authors	Description
Semantic-wise	Dong [38] Brosch [39] Shakeri [40] Zhenglun [41] Milletari [42] Raghav [43]	The main objective of the semantic-wise segmentation is to link each pixel of an image with its class label. It is called dense prediction because every pixel is predicted from the whole input image. Later, segmentation labels are mapped with the input image in a way that minimizes the loss function.
Patch–wise	Kamnitsas [44] Pereira [45] Havaei [46] Zhang [35] Brebisson [36] Moeskops [37]	Patch-wise segmentation handles high-resolution images, and the input images are split as local patches. An *N*×*N* patch is extracted from the input image. These patches are trained and provide class labels to identify normal or abnormal brain images. The design network consists of convolution layers, transfer functions, pooling, and sub-sampling layers, and fully connected layers.
Cascaded	Dou [47]	The cascaded architecture types are used to combine two different CNN architectures. The output of the first architecture is fed into the second architecture to get classification results. The first architecture is used to train the model with the initial prediction of class labels, and later for fine-tuning.
Single-modality	Moeskops [37] Brebisson [36] Raghav [43] Milletari [42] Shakeri [40]	This type of modality refers to single-source information and is adaptable to different scenarios. The single modality commonly used in the public dataset for tissue-type segmentation in brain MRI (mainly T1-W images).
Multi-modality	Zhang [35] Chen [48] Lyksborg [49]	Multi-source information can be used, and it might require a larger number of parameters than using a single modality. The advantage of using multi-modality is to gain valuable contrast information. Furthermore, using multi-path configurations, the imaging sequences can be processed in parallel (e.g., T1 and T2, fluid-attenuated inversion recovery (FLAIR)).

**Table 3:** The brain structure segmentation methods based on deep learning.

No.	Authors	Year	Strategies	Dimension	Key Method	Classifier	Dataset
1	Zhang [35]	2015	Patch-wise	2D	CNN	Soft-max	Clinical data
2	Brebisson [36]	2015	Patch-wise	2D/3D	CNN	Soft-max	MICCAI 2012
3	Moeskops [37]	2016	Patch-wise	2D/3D	CNN	Soft-max	NeoBrainS12
4	Bao [69]	2016	Patch-wise	2D	CNN	Soft-max	IBSR/LPBA40
5	Dong [38]	2016	Semantic-wise	2D	CNN	Soft-max	Clinical data
6	Shakeri [40]	2016	Semantic-wise	2D	FCNN	Soft-max	IBSR data
7	Raghav [43]	2017	Semantic-wise	2D/3D	M-Net + CNN	Soft-max	IBSR/ MICCAI 2012
8	Milletari [42]	2017	Semantic-wise	2D/3D	Hough-CNN	Soft-max	MICCAI 2012
9	Dolz [70]	2018	Semantic-wise	3D	CNN	Soft-max	IBSR/ABIDE
10	Wachinger [71]	2018	Patch-Based	3D	CNN	Soft-max	MICCAI 2012
11	Zhenglun [41]	2018	Semantic-wise	2D	Wavelet + CNN	Soft-max	Clinical data
12	Khagi [72]	2018	Semantic-wise	2D	SegNet + CNN	Soft-max	OASIS Dataset
13	Bernal [73]	2019	Patch-Based	2D/3D	FCNN	Soft-max	IBSR, MICCAI2012 & iSeg2017
14	Jiong [74]	2019	Semantic-wise	2D	U-net	Soft-max	MICCAI2017
15	Chen [75]	2019	Semantic-wise	2D	LCMV	-	BrainWeb
16	Pengcheng [76]	2020	Semantic-wise	3D/4D	Fuzzy C-mean	-	BLSA

**Table 4:** Overview of papers using deep learning techniques for the segmentation of brain MRI.

Authors	Methods	Application: Key Features
Zhang [35]	CNN	Tissue segmentation: multi-modal 2D segmentation for isointense brain tissues using the deep CNN architecture.
Brebisson [36]	CNN	Anatomical segmentation: fusing multi-scale 2D patches with a 3D patch using a CNN.
Moeskops [37]	CNN	Tissue segmentation: CNN trained on multiple patches and kernel sizes to extract information from each voxel.
Bao [69]	CNN	Anatomical segmentation: multi-scale late fusion CNN with a random walker as a novel label consistency method.
Dong [38]	CNN	Tissue segmentation: FCN with a late fusion method on different modalities.
Shakeri [40]	FCNN	Anatomical segmentation: FCN followed by Markov random fields, whose topology corresponds to a volumetric grid.
Raghav [43]	M-Net + CNN	Tissue segmentation: the 3D contextual information of a given slice is converted into a 2D slice using CNN.
Milletari [42]	Hough-CNN	Anatomical segmentation: Hough-voting to acquire mapping from CNN features to full patch segmentations.
Dolz [70]	CNN	Anatomical segmentation: 3D CNN architecture for the segmentation of subcortical MRI brain structure.
Wachinger [71]	CNN	Anatomical segmentation: neuroanatomy in T1-W MRI segmentation using deep CNN.
Zhenglun [41]	Wavelet + CNN	Tissue segmentation: pre-processing is performed with the wavelet multi-scale transformation, and then, CNN is applied for the segmentation of brain MRI.
Bernal [73]	FCNN	Tissue segmentation: the quantitative analysis of patch-based FCNN.
Jiong [74]	U-net	Tissue segmentation: skip-connection U-net for WM hyper intensities segmentation.
Chen [75]	LCMV	Tissue segmentation: new iterative linearly constrained minimum variance (LCMV) classification-based method developed for hyperspectral classification.
Pengcheng [76]	Fuzzy C-mean	Tissue segmentation: fuzzy C-means framework to improve the temporal consistency of adults’ brain tissue segmentation.

**Table 5:** Comparison of the state-of-the-art methods in the field of AD diagnosis.

No.	Authors	Year	Content	Modalities	Key Method	Classifier	Data (Size)
1	Siqi [81]	2014	Full brain	MRI	Auto-encoder	Soft-max	ADNI (311)
2	Suk [82]	2015	Full brain	MRI + PET	CNN	Soft-max	ADNI (204)
3	Payan [83]	2015	Full brain	MRI	CNN	Soft-max	ADNI (755)
4	Andres [84]	2016	Gray matter	MRI + PET	Deep Belief Network	NN	ADNI (818)
5	Hosseini [85]	2016	Full brain	fMRI	CNN	Soft-max	ADNI (210)
6	Saraf [86]	2016	Full brain	fMRI	CNN	Soft-max	ADNI (58)
7	Mingxia [87]	2017	Full brain	MRI	CNN	Soft-max	ADNI (821)
8	Aderghal [88]	2017	Hippocampus	MRI + DTI	CNN	Soft-max	ADNI (1026)
9	Shi [89]	2017	Full brain	MRI + PET	Auto-encoder	Soft-max	ADNI (207)
10	Korolev [90]	2017	Full brain	MRI	CNN	Soft-max	ADNI (821)
11	Jyoti [91]	2018	Full brain	MRI	CNN	Soft-max	OASIS (416)
12	Donghuan [92]	2018	Full brain	MRI	CNN	Soft-max	ADNI (626)
13	Khvostikov [93]	2018	Hippocampus	MRI + DTI	CNN	Soft-max	ADNI (214)
14	Aderghal [94]	2018	Hippocampus	MRI + DTI	CNN	Soft-max	ADNI (815)
15	Lian [95]	2018	Full brain	MRI	FCN	Soft-max	ADNI (821)
16	Liu [96]	2018	Full brain	MRI + PET	CNN	Soft-max	ADNI (397)
17	Lee [97]	2019	Full brain	MRI	CNN	Alex-Net	ADNI (843), OASIS (416)
18	Feng [98]	2019	Full brain	MRI + PET	CNN	Soft-max	ADNI (397)
19	Mefraz [99]	2019	Full brain	MRI	Transfer learning	Soft-max	ADNI (50)
20	Ruoxuan [100]	2019	Hippocampus	MRI	CNN	Soft-max	ADNI (811)
21	Ahmed [101]	2019	Full brain	MRI	CNN	Soft-max	ADNI (352) GARD (326)
22	Fung [102]	2020	Full brain	MRI + PET	CNN	Adaboost	ADNI (352)
23	Kam [103]	2020	Full brain	MRI	CNN	Soft-max	ADNI (352)
24	Shi [104]	2020	Full brain	MRI + PET + CSF	Machine learning	Adaboost	ADNI (202)

**Table 6:** Overview of existing methods using deep learning for the classification of AD.

Authors	Methods	Applications: Key Features
Siqi [81]	Auto-encoder	AD/HC classification: deep learning architecture contains sparse auto-encoders and a softmax regression layer for the classification of AD
Suk [82]	CNN	AD/MCI/HC classification: neuroimaging modalities for latent hierarchical feature representation from extracted patches using CNN
Payan [83]	CNN	AD/MCI/HC classification: 3D CNN pre-trained with sparse auto-encoders
Andres [84]	Deep Belief Network	AD/HC classification: automated anatomical labeling brain regions for the construction of classification techniques using deep learning architecture
Hosseini [85]	CNN	AD/MCI/HC classification: 3D CNN pre-trained with a 3D convolutional auto-encoder on MRI data
Saraf [86]	CNN	AD/HC classification: adapted Lenet-5 architecture on fMRI data
Mingxia [87]	CNN	AD/MCI/HC classification: landmark-based deep multi-instance learning framework for brain disease diagnosis
Aderghal [88]	CNN	AD/HC classification: separate CNN base classifier to form an ensemble of CNNs, each trained with a corresponding plane of MRI brain data
Shi [89]	Auto-encoder	AD/MCI/HC classification: multi-modal stacked deep polynomial networks with an SVM classifier on top layer using MRI and PET
Korolev [90]	CNN	AD/MCI/HC classification: residual and plain CNNs for 3D brain MRI
Jyoti [91]	CNN	AD/HC classification: deep CNN model for resolving an imbalanced dataset to identify AD and recognize the disease stages.
Donghuan [92]	CNN	AD/MCI classification: early diagnosis of AD by combing the multiple different modalities using multiscale and multimodal deep neural networks.
Khvostikov [93]	CNN	AD/HC classification: multi-modality fusion on hippocampal ROI using CNN
Aderghal [94]	CNN	AD/HC classification: diffusion tensor imaging modality from MRI using the transfer learning method
Lian [95]	FCN	AD/MCI/HC classification: CNN to discriminate the local patches in the brain MRI and multi-scale features are fused to construct hierarchical classification models for the diagnosis of AD.
Liu [96]	CNN	AD/MCI/HC classification: CNN to learn multi-level and multimodal features of MRI and PET brain images.
Lee [97]	CNN	AD/MCI/HC classification: data permutation scheme for the classification of AD in MRI using deep CNN.
Feng [98]	CNN	AD/MCI/HC classification: 3D-CNN designed to extract deep feature representation from both MRI and PET. Fully stacked bidirectional long short-term memory (FSBi-LSTM) applied to the hidden spatial information from deep feature maps to improve the performance.
Mefraz [99]	Transfer learning	AD/MCI/HC classification: transfer learning with intelligent training data selection for the prediction of AD and CNN pre-trained with VGG architecture.
Ruoxuan [100]	CNN	AD/MCI/HC classification: a new hippocampus analysis method combining the global and local features of the hippocampus by 3D densely connected CNN.
Ahmed [101]	CNN	AD/HC classification: ensembles of patch-based classifiers for the diagnosis of AD.
Fung [102]	CNN	AD/MCI/HC classification: an ensemble of deep CNNs with multi-modality images for the diagnosis of AD.
Kam [103]	CNN	AD/MCI/HC classification: CNN framework to simultaneously learn embedded features from brain functional networks (BFNs).
Shi [104]	Machine Learning	AD/MCI/HC classification: MRI, PET, and CSF are used as multimodal data. Coupled boosting and coupled metric ensemble scheme to model and learn an informative feature projection form the different modalities.

**Table 7:** Summary of the validation measures of brain segmentation and their mathematical formula regarding the number of true positives (TP), false positives and false-negative (FN) at voxel and lesion levels (TPL, FPL, and FNL).

Metrics of Segmentation Quality	Mathematical Description
True positive rate, TPR (Sensitivity)	$TPR=\frac{TP}{TP+FN}$
Positive predictive rate, PPV (Precision)	$PPV=\frac{TP}{TP+FP}$
Negative predictive rate, NPV	$\frac{TN}{TN+FN}$
Dice similarity coefficient, DSC	$TPR=\frac{2TP}{2\;TP+\;\mathrm{FP}\;+FN}$
Volume difference rate, VDR	$TPR=\frac{\left|FP-FN\right|}{TP+FN}$
Lesion-wise true positive rate, LTPR	$LTPR=\frac{TPL}{TPL+FNL}$
Lesion-wise positive predictive value, LPPV	$TPPV=\frac{TPL}{TPL+FPL}$
Specificity	$\frac{TN}{TN+FP}$
F1 score	$\frac{2\;TP}{2\;TP+\;\mathrm{FP}\;+FN}$
Accuracy	$\frac{TP+TN}{TP+\;\mathrm{TN}\;+\;\mathrm{FP}\;+FN}$
Balanced Accuracy	$\frac{\left(Sensitivity+Specificity\right)}{2}$

**Table 8:** Summary of results in the existing methods using deep learning approaches for brain structure segmentation. (†: DSC, *: JI) (Unit: %).

Authors		MICCAI [17]			OASIS [15]			Clinical/IBSR [18]		
		DSC and JI			DSC and JI			DSC and JI		
		CSF	GM	WM	CSF	GM	WM	CSF	GM	WM
1	Zhang [35]	-	-	-	-	-	-	83.5 ^†^	85.2 ^†^	86.4 ^†^
2	Brebisson [36]	72.5 ^†^	72.5 ^†^	72.5 ^†^	-	-	-	-	-	-
3	Moeskops [37]	73.5 ^†^	73.5 ^†^	73.5 ^†^	-	-	-	-	-	-
4	Bao [69]	-	-	-	-	-	-	82.2 ^†^	85.0 ^†^	82.2 ^†^
5	Dong [38]	-	-	-	-	-	-	85.5 ^†^	87.3 ^†^	88.7 ^†^
6	Zhenglun [41]	-	-	-	-	-	-	94.3 *	90.2 *	91.4 *
7	Khagi [72]	-	-	-	72.2 ^†^ 73.0 *	74.6 ^†^ 74.0 *	81.9 ^†^ 85.0 *	-	-	-
8	Shakeri [40]	-	-	-	-	-	-	82.4 ^†^	82.4 ^†^	82.4 ^†^
9	Raghav [43]	74.3 ^†^	74.3 ^†^	74.3 ^†^	-	-	-	84.4 ^†^	84.4 ^†^	84.4 ^†^
10	Milletari [42]	-	-	-	-	-	-	77.0 ^†^	77.0 ^†^	77.0 ^†^
11	Dolz [70]	-	-	-	-	-	-	90.0 ^†^	90.0 ^†^	90.0 ^†^
12	Wachinger [71]	90.6 ^†^	90.6 ^†^	90.6 ^†^	-	-	-	-	-	-
13	Chen [75]	-	-	-	-	-	-	93.6 ^†^	94.8 ^†^	97.5 ^†^

**Table 9:** A brief review of the state-of-the-art methods for AD classification (AD vs. HC) and MCI conversion prediction (pMCI vs. sMCI) using MRI data. (The best results obtained for different metrics are highlighted in bold).

Authors		Subjects	AD vs. HC				pMCI vs. sMCI			
			ACC	SEN	SPE	AUC	ACC	SEN	SPE	AUC
1	Siqi [81]	204HC + 180AD	0.79	0.83	0.87	0.78	-	-	-	-
2	Suk [82]	101HC + 128sMCI + 76pMCI + 93AD	0.92	0.92	0.95	**0.97**	0.72	0.37	**0.91**	0.73
3	Korolev [90]	61HC + 77sMCI + 43pMCI + 50AD	0.80	-	-	0.87	0.52	-	-	0.52
4	Khvostikov [93]	58HC + 48AD	0.85	0.88	0.90	-	-	-	-	-
5	Lian [95]	429HC + 465sMCI + 205pMCI + 358AD	0.90	0.82	0.97	0.95	**0.81**	0.53	0.85	**0.78**
6	Mingxia [87]	229HC + 226sMCI + 167pMCI + 203AD	0.91	0.88	0.93	0.95	0.76	0.42	0.82	0.77
7	Andres [84]	68HC + 70AD	0.90	0.86	0.94	0.95	-	-	-	-
8	Adherghal [84]	228HC + 188AD	0.85	0.84	0.87	-	-	-	-	-
9	Donghuan [92]	360HC + 409sMCI + 217pMCI	-	-	-	-	0.75	**0.73**	0.76	-
10	Shi [89]	52 NC + 56 sMCI + 43 pMCI + 51AD	0.95	0.94	0.96	0.96	0.75	0.63	0.85	0.72
11	Payan [83]	755 subjects (AD, MCI, HC)	0.95	-	-	-	-	-	-	-
12	Hosseini [85]	70HC + 70AD	**0.99**	-	**0.98**	-	-	-	-	-
13	Lee [97]	843 subjects (AD, MCI, HC)	0.98	0.96	0.97	-	-	-	-	-
14	Liu [96]	397 subjects (AD, MCI, HC)	0.93	0.92	0.93	0.95	-	-	-	-
15	Feng [98]	397 subjects (AD, MCI, HC)	0.94	**0.97**	0.92	0.96	-	-	-	-
16	Ruoxuan [100]	811 subjects (AD, MCI, HC)	0.90	0.86	0.92	0.92	0.73	0.69	0.75	0.76

**Table 10:** Summary of the hardware and software details required for the segmentation of brain MRI and classification of AD using deep learning methods.

Author	Dataset	Scanner	Hardware	Software	Training Time
Zhang [35]	Clinical data	3T Siemens	Tesla K20c GPU with 2496 cores	iBEAT toolbox ITK-SNAP toolbox	less than one day
Brebisson [36]	MICCAI 2012	-	NVIDIA Tesla K40 GPU with 12 GB memory.	Python with Theano framework	-
Moeskops [37]	NeoBrainS12	3T Philips Achieva	-	BET toolbox FMRIB software library	-
Bao [69]	IBSR LPBA40	1.5 T GE	-	FLIRT toolbox	-
Dong [38]	Clinical data	3T Siemens	-	Python with Caffe framework iBEAT toolbox	-
Raghav [43]	IBSR MICCAI 2012 LPBA40 Hammers67n20	- - 1.5 T GE 1 T Philips HPQ	NVIDIA K40 GPU, with 12 GB of RAM.	Python with Keras packages BET toolbox	-
Milletari [42]	Clinical data	-	Intel i7 quad-core workstations with 32 GB of RAM and Nvidia GTX 980 (4 GB -RAM).	Python with Caffe framework	-
Dolz [70]	IBSR ABIDE	-	Intel(R) Core(TM) i7-6700 K 4.0 GHz CPU and NVIDIA GeForce GTX 960 GPU with 2 GB of memory.	Python with Theano framework FreeSurfer 5.1 tool Medical Interaction Tool Kit	2 days and a half
Wachinger [71]	MICCAI 2012	-	NVIDIA Tesla K40 and TITAN X with 12 GB GPU memory	Python with Caffe framework FreeSurfer tool	1 day(train) 1 h(test)
Bernal [73]	IBSR MICCAI2012 iSeg2017	-	Ubuntu 16.04, with 128 GB RAM and TITAN-X PASCAL GPU with 8 GB RAM	Python with Keras packages	-
Jiong [74]	MICCAI2017	-	Ubuntu 16.04 with 32 GB RAM and GTX 1080 Ti GPUs.	Python with Keras packages	-
Chen [75]	BrainWeb	1.5 T Siemens	Windows 7 computer with CPU Intel R Xeon R E5-2620 v3 @ 2.40 GHz processor and 32 GB RAM	-	-
Pengcheng [76]	BLSA	-	-	FSL software 3D-Slicer	-
Hosseini [85]	ADNI	1.5 T Siemens Trio	Amazon EC2 g 2.8 x large with GPU GRID K520	Python with Theano framework	-
Saraf [86]	ADNI	3T Siemens Trio	NVIDIA GPU	Python with Caffe framework BET toolbox FMRIB Software Library v 5.0	-
Mingxia [87]	ADNI-1 ADNI-2 MIRIAD	1.5 T Siemens Trio 3 T Siemens Trio 1.5 T Signa GE	NVIDIA GTX TITAN 12 GB GPU	MIPAV software FSL software	27 h <1 s (test)
Aderghal [88]	ADNI	1.5 T Siemens Trio	Intel^®^ Xeon^®^ CPU E5-2680 v2 with 2.80 GHz and Tesla K20Xm with 2496 CUDA cores GPU	Python with Caffe framework	2 h, 3 min
Jyoti [91]	OASIS	1.5 T Siemens	Linux X86-64 with AMD A8 CPU, 16 GB RAM and NVIDIA GeForce GTX 770	Python with Tensorflow and Keras library	-
Khvostikov [93]	ADNI	1.5 T Siemens Trio	Intel Core i7-6700 HQ with Nvidia GeForce GTX 960 M and Intel Core i7-7700 HQ CPU with Nvidia GeForce GTX 1070 GPU	Python with Tensorflow framework BET toolbox	-
Lian [95]	ADNI-1 ADNI-2	1.5 T Siemens Trio 3 T Siemens Trio	NVIDIA GTX TITAN 12 GB GPU	Python with Keras packages	-
Liu [96]	ADNI	1.5 T Siemens Trio	GPU NVIDIA GTX1080.	Python with Theano framework and Keras packages	
Lee [97]	ADNI OASIS	1.5 T Siemens Trio 1.5 T Siemens	Nvidia GTX 1080Ti GPU	-	-
Feng [98]	ADNI	1.5 T Siemens Trio	Windows with NVIDIA TITA- Xt GPU	MIPAV Software Keras library with Tensorflow as backend	-
Ruoxuan [100]	ADNI	1.5 T Siemens Trio	Ubuntu14.04-x64/ GPU of NVIDIA GeForce GTX 1080 Ti	FreeSurfer tool Keras library with Tensorflow as backend	-
Ahmed [101]	ADNI GARD	1.5 T Siemens Trio	Intel(R) Xeon (R) CPU E5-1607 v4 @ 3.10 GHz, 32 GB RAM NVIDIA Quadro M4000	Keras library with Tensorflow as backend	-
Fung [102]	ADNI	1.5 T Siemens Trio	Desktop PC equipped with Intel Core i7, 8 GB memory and GPU with 16 G NVIDIA P100 × 8	Ubuntu 16.04, Keras library with Tensorflow MATLAB 2014b with SPM	-
